# In-Situ X-ray Imaging of Sublimating Spin-Frozen Solutions

**DOI:** 10.3390/ma13132953

**Published:** 2020-07-01

**Authors:** Wannes Goethals, Brecht Vanbillemont, Joris Lammens, Thomas De Beer, Chris Vervaet, Matthieu N. Boone

**Affiliations:** 1 Department of Physics and Astronomy, Radiation Physics, Ghent University, Proeftuinstraat 86/N12, B-9000 Gent, Belgium; matthieu.boone@ugent.be; 2 Centre for X-ray Tomography (UGCT), Ghent University, Proeftuinstraat 86, B-9000 Gent, Belgium; 3 Laboratory of Pharmaceutical Process Analytical Technology (LPPAT), Department of Pharmaceutical Analysis, Ghent University, Ottergemsesteenweg 460, B-9000 Gent, Belgium; brecht.vanbillemont@ugent.be (B.V.); thomas.debeer@ugent.be (T.D.B.); 4 Laboratory of Pharmaceutical Technology, Department of Pharmaceutics, Ghent University, Ottergemsesteenweg 460, B-9000 Gent, Belgium; joris.lammens@ugent.be (J.L.); chris.vervaet@ugent.be (C.V.)

**Keywords:** X-ray micro-CT, dynamic imaging, in-situ imaging, spin-freezing, freeze-drying

## Abstract

Spin-freeze-drying is a promising technique to enable long-term storage of pharmaceutical unit doses of aqueous drug solutions. To investigate the sublimation of the ice during the primary phase of freeze-drying, X-ray imaging can yield crucial temporally resolved information on the local dynamics. In this paper, we describe a methodology to investigate the sublimation front during single unit-dose freeze-drying using 4D in-situ X-ray imaging. Three spin-frozen samples of different solutions were imaged using this methodology and the process characteristics were analysed and reduced to two-dimensional feature maps.

## 1. Introduction

Freeze-drying or lyophilisation is a low-temperature drying process to stabilize heat-sensitive bio-pharmaceutical materials for storage and distribution. By accurately controlling the heat transfer and pressure, water is separated from an aqueous solution in three consecutive steps to achieve a dry stable cake: Freezing, primary drying and secondary drying [1,2,3]. While freeze-drying in industry is typically approached as a batch process, spin-freeze-drying is a promising technique for continuous freezing of unit doses in pharmaceutical sciences [4,5]. Single cylindrical vials are spun rapidly around their longitudinal axis while a stream of cold sterile gas is sent around the vial. Since the surface area of the frozen substance is spread over the vial’s wall as a thin layer, this allows for a significantly higher sublimation rate during the drying steps as compared with the traditional technique and results in decreased variations from vial to vial. These intra-vial fluctuations of the sublimation process can be monitored on a single vial level using thermal imaging [6,7,8]. High-resolution X-ray computed microtomography (micro-CT) is an increasingly popular technique to visualize objects and structures in 3D. This non-destructive technique, which requires relatively little sample preparation, is commonly being used in materials sciences [9], geosciences [10], additive manufacturing [11], biology, etc. In recent years, a significant effort is spent on extending this technique towards time as a 4th dimension. Indeed, the non-destructive nature of X-ray imaging is a great asset in studying dynamic processes as they take place. This technique, often named 4D micro-CT, has been applied already in a large number of application areas, such as petrophysics [12,13] and geosciences [14,15]. Within 4D micro-CT, a great variety exists in temporal and spatial resolution. For slower processes as well as induced processes such as compression, time-lapse imaging can be a valid approach, e.g., in pharmaceutical drug delivery [16], geosciences [17], biology [18,19], etc. While time-lapse imaging should in principle be free of motion artefacts, settlement of the material often hinders proper imaging or affects the dynamic process itself, if it is even feasible to stop the process temporarily. For these reasons, it is in many cases better to perform continuous scanning. In this acquisition scheme, radiographs are acquired continuously while the rotating spans a (large) number of full rotations. From this data, an arbitrary number of 3D volumes can be reconstructed using standard reconstruction techniques, each corresponding to a certain time interval and attributed to a certain point in time (usually the middle of the interval). While motion artefacts are inherently present, this acquisition scheme allows for correction methods [20,21] and the possibility for faster scanning allows to minimize these artefacts. This scheme is particularly useful for very fast scanning, e.g., in materials science [22] and can also be combined with gating to investigate even faster processes [23].

In many cases, the dynamic processes that are under investigation require peripheral equipment to induce them. This can be e.g., fluid flow cells [24], cooling stages [25], heating stages [26], stirring equipment [27], squeezing systems [28] etc. Imaging a dynamic process in-situ or even operando poses several constraints on the peripheral equipment, as it should be compatible with the X-ray imaging. The most important constraint is the transparency of the equipment to X-rays, at least at the position of the sample. This strongly limits the amount of material that can be used in such peripheral equipment. Furthermore, particularly in lab-based imaging, the diameter of the equipment at the region under investigation should be sufficiently small, as geometric magnification typically requires a small distance between the source and the center of the object. While this is often inherently the case due to the former transparency requirement, this is in some cases a very stringent additional requirement. This is notably the case in temperature stages, which require sufficient thermal insulation from the outside air and the X-ray source. Lastly, it needs to be mounted on the scanning stage. As in many micro-CT setups, and notably all synchrotron setups, the object needs to be rotated, sometimes at high speed, there is not always a straightforward implementation. In lab-based imaging, gantry-based setups exist which do not require the rotation of this object [29,30], hence drastically simplifying the design and cost of the peripheral equipment.

One of the major challenges of 4D micro-CT is the analysis of this vast amount of data. Indeed, such data can no longer be clearly visualized and data reduction is necessary to enable quantitative interpretation. Due to the variety of applications of 4D micro-CT, a generic methodology cannot be set up. While in some cases a visual assessment of the process is made based on 3D visualizations for a selection of a few time steps [12], such methods can also be combined with 3D analysis to yield a single parameter per pore or grain in the dataset [31].

In the research field of lyophilisation, micro-CT has already proven to be a viable technique to assess the intra-vial and inter-vial variability of the cake structure and to detect product defects by imaging the cake structures after freeze-drying [32,33,34,35]. This 3D imaging methodology provides important information when new sublimation techniques need to be investigated, yet the dynamics of the cake formation remain poorly investigated. In subsequent analysis, the dried product mass transfer resistance is often calculated based on the pore diameter. A one-dimensional sublimation model is assumed, while it is known that the batch freeze-drying process displays multiple sublimation fronts. In this paper, we present a methodology for operando X-ray imaging of spin-freezing of pharmaceutical samples and analyzing the sublimation front in this process. This sublimation front, which is the interface between the dry layer and the ice layer, shows small variations at each time of the primary drying phase. As such, its structural and temporal variations reveal the local properties that drive the sublimation. Using a state-of-the-art rotating gantry micro-CT setup, dedicated peripheral equipment and custom designed analysis software, we were able to quantify the sublimation properties and represent it in 2D images to gain insight into the sublimation process of single unit-dose freeze-drying.

## 2. Materials and Methods

### 2.1. Spin-Freezing Materials and Method

The setup and imaging methodology is evaluated based on three different formulations. The first formulation contained 4% bovine serum albumin (BSA), the second had 3% (*w/V*) mannitol solution in deionised water. As a reference, the third sample was plain deionised water without dissolved substance. The two solutions have a high collapse temperature ($T_{c}$) (−9 °C for BSA and −2 °C for mannitol) [36], which minimizes the risk of (micro-)collapse during the dynamic imaging experiments. These solutions were chosen for their different structure in solid state, being amorphous for the BSA formulation and mainly crystalline for mannitol. All materials were purchased from Sigma Aldrich (Zwijnaarde, Belgium).

For each formulation, 3.5 mL was transferred in a 10R Fiolax^®^ clear Schott glass vial. This vial was positioned vertically in a single vial spin-freezer (Rheavita, Zwijnaarde, Belgium), where it was rotated at 2900 rpm around its longitudinal axis while being frozen with cold compressed air (130 L/min, −60 °C) within 6 min. The temperature of the vial was measured using a FLIR A655sc thermal infrared camera (Thermal focus, Ravels, Belgium) to assure that the temperature was well below −45 °C. This temperature setting was chosen to assure that full solidification was obtained, with a safe margin for variations within the sample. Finally, the vial was stored on dry ice (temperature below −78.5 °C) and transported within the same day to the micro-CT scanning facility. Vials were sealed with a 22 mm siliconized bromobutyl lyophilisation stopper (West Pharma, Exton, PA, USA) during storage and transport.

### 2.2. Micro-CT Setup

The thickness of the frozen product layer was monitored during primary drying via micro-CT scanning. Due to the number of wires and tubes connected to the peripheral equipment, and notably the vacuum system (see below), a rotating-gantry micro-CT system was selected for this study. The system, dubbed the Environmental Micro-CT (EMCT) scanner [29], consists of a rotating gantry with central bore of approximately 19 cm diameter, which is mounted on a granite table with an identical bore on a height of approximately 1 m above the ground level. On top of the gantry, a platform carries the control PC and a linear translation stage, on which both the X-ray source and detector are mounted. The X-ray source is a L9181-02 closed-type directional tube with a minimal focal spot size of approximately 5 μm (Hamamatsu Photonics, Hamamatsu City, Japan). The X-ray detector is a Xineos-1313 flat-panel with $100^{2}$ μm${}^{2}$ pixels (Teledyne DALSA, Waterloo, ON, Canada). The distance between source and detector is fixed at 365.7 mm, yet using a motorized translation stage on which the X-ray source and detector are mounted, the geometrical magnification can be changed. For these experiments, the tube was operated at 90 kVp and 9 W target power (macrofocus spot mode). With a source-to-object distance of 109.7 mm, a geometrical magnification of 3.333 was achieved, resulting in a projected pixel size of $30^{2}$ μm${}^{2}$.

The scanner rotated continuously around the spin-frozen vial while recording. A full vial scan (360°) was made each 176.11 s during primary drying, recording 1600 projections per rotation. The process was imaged during 40 (BSA), 35 (mannitol) or 30 (water) sequential rotations in total (i.e., 117, 103 and 88 min of primary drying, respectively). From this data, 40 (BSA), 69 (mannitol) and 30 (water) 3D volumes were reconstructed at a time interval of 2.93 min (BSA and water) and 1.47 min (mannitol).

### 2.3. Freeze-Drying Peripheral Equipment

**Figure 1:**
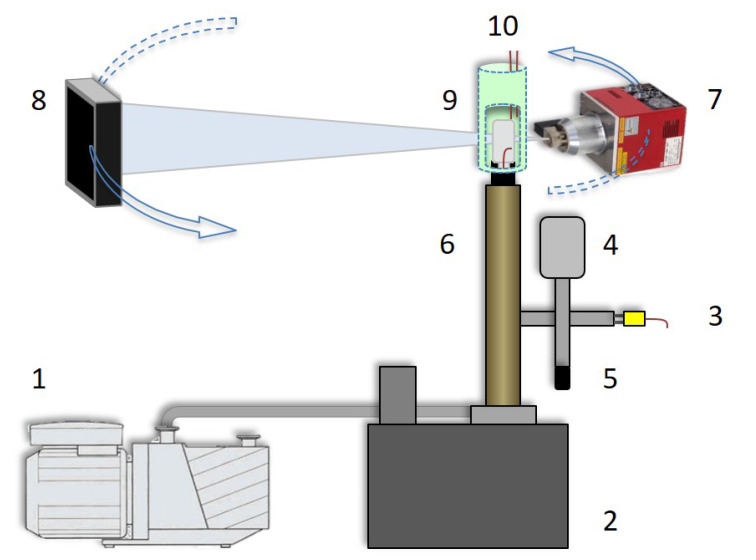
Schematic of the setup dedicated to visualizing the primary drying phase of single unit-dose samples. 1: Vacuum pump; 2: Condensor; 3: Frozen product thermocouple read-out; 4: Proportional pressure valve; 5: Pirani gauge; 6: Vial manifold; 7: X-ray source; 8: Detector; 9: Spin-frozen vial; 10: Heating pad wrapped with isolating fabric.

Figure 1 shows the setup that was installed on the EMCT scanner for this single-vial dynamic drying scan. The spin-frozen vial was mounted top down on the vial manifold, and the sublimated water vapour was evacuated to the condenser underneath the micro-CT scanner. A Polytetrafluoroethylene (PTFE) O-ring ensured an integral vacuum connection between the vial neck and the manifold. A DS102 rotational vacuum pump (Agilent technologies, Leini, Italy) was connected to the condenser and the pressure of the system was monitored by a VD84 Pirani gauge (Thyracont vacuum instruments, Passau, Germany). Dry nitrogen gas was used for controlling the pressure at 5 Pa using the EVR116 proportional pressure valve (Pfeiffer Vacuum, Asslar, Germany) and proportional–integral–derivative (PID) controllers.

The heat flux required for sublimation was provided by a polyester electric heating foil (Thermo TECH, RohrBach, Germany) wrapped tightly around the glass vial. This heating pad was surrounded by a low attenuating insulating poly urethane fabric to prevent additional influence by the environment. The temperature of the heating pad was fixed at 40 °C for the BSA and water sample, and at 60 °C for the mannitol sample, while the temperature was monitored at three locations. The temperature of the ice layer was measured with a thin gauge type-K thermocouple (Labfacility, Leeds, United Kingdom) located in the ice surface near the vial neck. The temperature of the heating pad and the glass vial were measured at the top of the heating pad and by a thermocouple attached with a circular copper disk to the bottom of the vial’s outer wall for a homogeneous readout. The rotating-gantry design of the EMCT scanner allowed for relatively low-cost peripheral equipment.

### 2.4. Cylindrical Vial Pose Estimation

As mentioned above, the vials have a cylindrical shape. Hence the resulting spin-frozen layer has a nearly cylindrical shape, with minor variations due to gravitational effects and the local composition during freezing. The projection series consisting of multiple full rotations was reconstructed to a time sequence of 3D volumes in a cylindrical coordinate system following the vial’s symmetry axis in order to ease further analysis [36]. Each single reconstruction depicts the state of the object within the quasi-static time frame in the sliding projection window.

**Figure 2:**
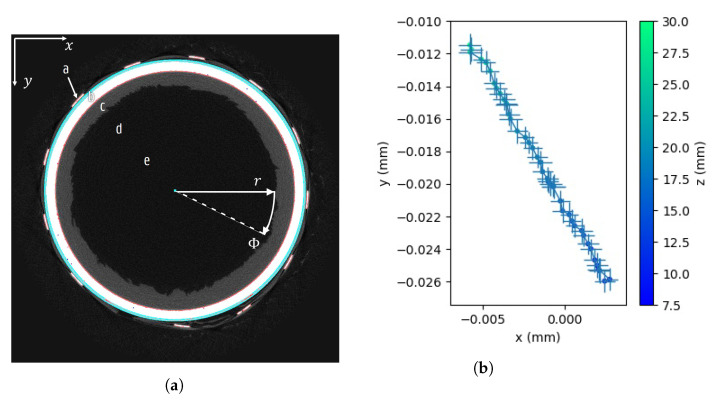
A Cartesian reconstruction was used to locate the vial’s axis for the final reconstruction in aligned cylindrical coordinates. (**a**) The following regions are present in the sample: The ceramic temperature mat (a), the glass vial (b), the ice layer (c), the invisible dry layer (d), and the central vacuum region (e). The segmented vial is coloured white, its real edges red and the detected circle and its centre in cyan. (**b**) A straight line was fitted through the measured circle centres at various heights to retrieve the vial’s axis.

In order of ascending radial distance, the following regions were present (Figure 2a): The inner vacuum, the dry cake layer, the sublimating frozen layer and the surrounding vial, heating and insulation system. In light of the investigation of the sublimation processes, the two material interfaces to the dry layer were important. However, the minimal contrast between the inner vacuum and the low-density dry layer inhibited the visualization of this layer. Therefore, all information had to be retrieved from the interface between the dry layer and the frozen layer. The shape of the sublimation front and the sublimation speed revealed intrinsic local properties of the frozen layer, which will be explained in more detail below.

Since the vials are generally not perfectly centered and aligned, a single initial volume was reconstructed for each sample to retrieve the vial’s position and orientation using a volumetric pose estimation technique. Such virtual alignment is less time-consuming than aligning the object and the rotation axis perfectly before scanning, particularly considering this alignment should be performed for each sample, while the mounting time should be kept minimal to minimize the effects on the dynamic process under investigation. The volumetric pose estimation technique is illustrated in Figure 2. At 40 different heights of the reconstruction, horizontal slices were segmented to extract the glass vial. Fake or non-circular edges due to noise were discarded by retaining only those 10% with the highest radial gradient w.r.t. the image centre in the reconstruction. In order to detect the centres of the circular edges corresponding to this vial, a randomized Hough transform (RHT) [37] was used using 5000 random edge triplets. The most significant circle was selected per slice, using the count rate as a weight. Through these centres, the axis of the vial was fitted by calculating the weighted covariance, yielding the pose of the sample.

The reconstructions should not have any dynamic global motion when the local sublimation is analysed, as the interference leads to false conclusions about the sublimation direction. This can be assured by either a very stable setup at all time scales, or by virtually correcting the motion before initiating the local analysis. The precision of the pose estimation was measured by performing pose estimations at every of the 40 time steps of the BSA scan that were reconstructed on a 1312 × 1016 × 1016 zyx grid with a spacing of 30 μm. These measurements were performed four times, capturing the variations due to the random nature of the RHT. Additionally, the dynamic fluctuations of the setup over the total duration of the scan were characterised.

### 2.5. Cylindrical CT Reconstruction and Image Analysis

To avoid resampling artefacts due to post-reconstruction alignment and conversion to cylindrical coordinates, the reconstructions were performed directly on the aligned cylindrical grid using the in-house developed framework CTrex [38].

The cylindrical volume was defined on a basis of aligned coordinates r,Φ,h with *r* being the distance to the vial’s axis, Φ the azimuthal angle and *h* the height coordinate. The ray tracing methods in the iterative reconstruction technique were adapted to this coordinate transformation [39]. The number of azimuthal divisions was selected such that the azimuthal dimension of the wedge-shaped voxels was equal to the radial dimension at the outer edge of the sample. Not surprisingly, this corresponded to the number of projections in each 360° rotation. The constant radial and height dimensions of the voxels were chosen equal to the Cartesian voxel size of 30 μm.

To facilitate further characterization of the inner sample surface, the 3D volume was reduced to feature maps in vertical hΦ projections, as the surface could be characterized by a limited number of values at each point (h,Φ). In the cases presented here, the density of the cake layer was too low to observe, and only the homogeneous ice material could be segmented. For each (h,Φ) coordinate, the corresponding ice layer thickness was therefore the main parameter of interest. Additionally, the presence and size of cavities or vertically or azimuthally extending intrusions were of interest and could be plotted. These feature maps allowed to perceive larger scale structures (e.g., cracks and channels) that could not be easily observed in the xyz coordinate datasets.

**Figure 3:**
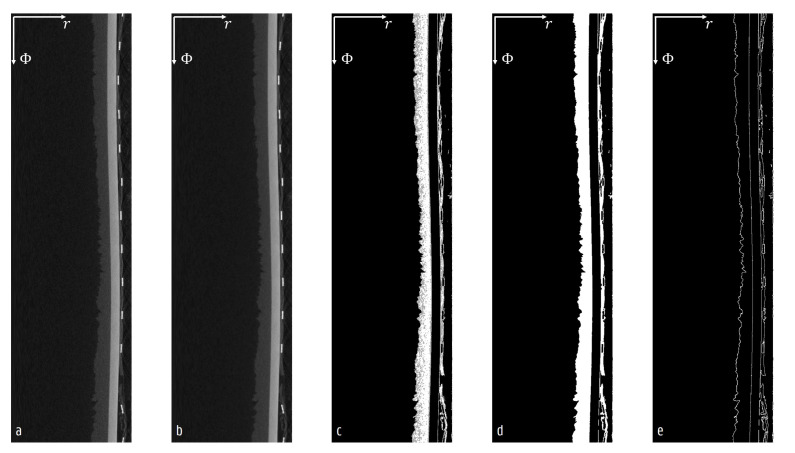
The full resolution cylindrical reconstruction (**a**) was processed using a series of 3D image operators (**b**–**e**) to locate the inner wall of the ice layer; (**b**) used a bilateral filter to smooth the image; (**c**) shows the two phases, between the hard and soft grey value limits, of the dual threshold segmentation in white and grey. In (**d**), a median filter was used to remove isolated voxels from the segmentation; (**e**) localizes the edges of the segmented ice layer.

The frozen layer was segmented from the cylindrical reconstruction as follows (Figure 3): First a bilateral filter was applied to reduce noise while maintaining the edges. Secondly, a dual threshold filter was used to distinguish the frozen layer from the other regions. Thirdly, a 3D median filter removed small spots resulting from residual noise while preserving the edges. Finally, an inner edge was extracted from the segmented image. Additionally, a Sobel-Feldman filter with a 3×3×3 kernel size indicated the surface’s horizontal and vertical angles with respect to the radial axis. Regions around the peripheral sensors and the narrowing sides of the vial were excluded from the analysis.

Analysing the filtered reconstructions, multiple measures were derived, one of which is the thickness of the ice layer $L_{ice}$. This was calculated as the radial distance between the first and the last segmented voxel at each position (h,Φ). Possible intrusions could be noticed by comparing this radial distance with the number of voxels contained in the ice layer along the radial direction. An additional important measure is the surface area at each location. This is affected by the radial position and local slope of the surface. While the frozen layer is sublimating, the radial distance gradually increases, offering more contact to the surrounding dry layer. Next to that, locations with steep inclinations offer an analogue increase of contact area. In this regard, the surface angles indicate the favourable sublimation directions. The characteristic measures were analyzed in this study from a more general point of view, without quantitatively comparing the results with the models.

## 3. Results

### 3.1. Pose Estimation Stability

**Figure 4:**
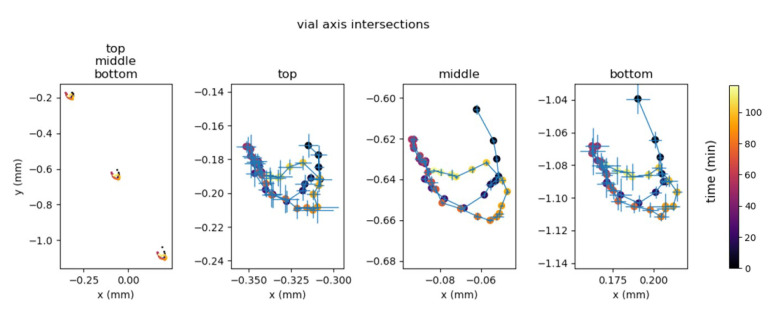
Vial axis intersections were measured at various heights within the reconstruction to estimate the pose stability.

The vial axis pose was determined four times for all 40 time steps of the BSA scan. The intersections of the axis through the top, middle and bottom slices are depicted in Figure 4, with coordinates relative to the slice centre. The error bars on the three detail axes of the figure depict the precision of each axis measurement. The measured standard deviation is 8.6 μm at the top, 8.1 μm at the bottom and 2.5 μm in the middle, which is well below the voxel size. The global spread through time reaches up to 60 μm, which is equal to two voxels. The color of the markers in Figure 4, which indicates the starting time of the dataset acquisition, reveals that the axis was translating back and forth rather than tilting throughout time, and that the displacement of the vial was still smaller between subsequent time steps than this spread of two voxels. As such, the error is believed to be due to a physical effect rather than a measurement error due to the method itself.

### 3.2. Surface Characteristics

**Figure 5:**
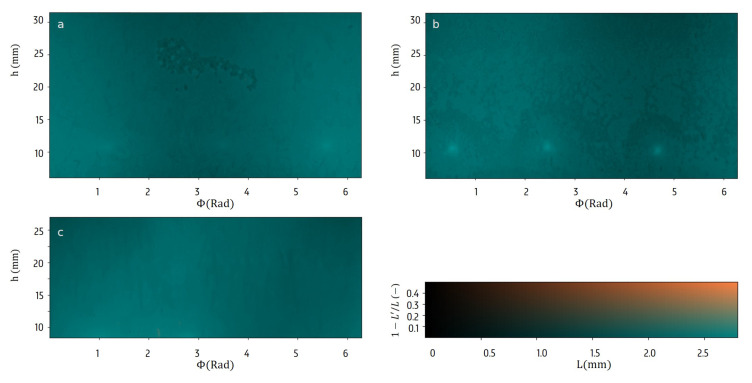
The surface of the ice layer at the initial time can be characterized mainly by a single thickness *L* for each location (Φ,h) in the samples with bovine serum albumin (BSA)hlBSA solution (**a**), mannitol solution (**b**) and water (**c**).

In Figure 5 the initial state of the ice layer surface is displayed by the thickness $L_{ice}\left(0\right)$ and the relative difference of $L_{ice}^{\prime }\left(0\right)$. It shows that the frozen layer is already affected by the formulation in the initial period. The water surface is relatively smooth, but in the mannitol formulation many small plateaus can be noticed. Three remarkable circular dots at a height of 10 mm can be noticed in the mannitol sample, and to a smaller extent in the BSA solution. These are possibly related to the position of the grippers in the spin-freezing stage, used to hold the vial while rotating. The disrupted contact with the cold gas affected the freezing characteristics in the initial step of the freeze-drying process.

**Figure 6:**
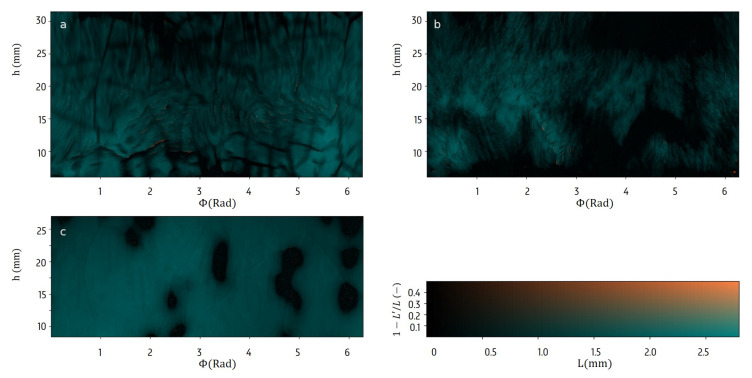
The ice surface after 70.3 min. The surface can exhibit multiple sublimation interfaces along the radial axis, which shows as a deviating integrated ice layer thickness $L^{\prime }$ coloured orange in these figures. For bovine serum albumin (BSA) (**a**) and mannitol (**b**), minor cavities can be noticed, no irregularities were noticed for the water sample (**c**).

70.3 min into the scan, the surface has evolved to Figure 6, with many local variations in the thickness *L*. Apparently, the sublimation rates differed, probably due to local structural and chemical variations of the frozen and dry layer. Next to that, non-radial sublimation has formed some small cavities at locations with high inclinations w.r.t. the radial axis. Larger-scale stable features like cracks or gaps also appear during the sublimation, which is crucial information that cannot be observed in a static scan after the process.

Significant differences can be observed between the sublimation front structures of the BSA, mannitol and water sample. While the BSA sample contains extended cracks with widths of about 1 mm running predominantly horizontally or vertically mixed with smaller wall structures, the mannitol sample has a surface with mainly smaller spikes. The ice surface in the water sample is very regular, with larger smoothly rounded holes. These differences between the three samples indicate that the chemical properties of the solution and the structure of the dry cake have a major impact on the freeze-drying process. Therefore it is important to investigate how these surface features correlate with the sublimation characteristics.

### 3.3. Sublimation Characteristics

**Figure 7:**
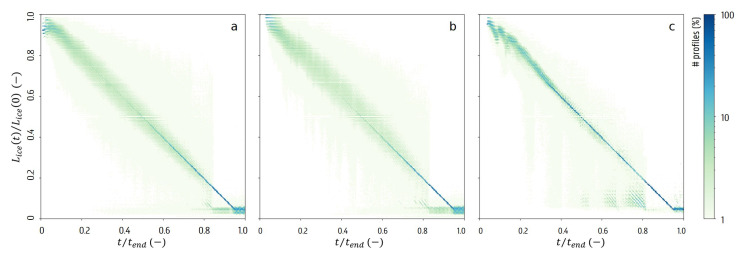
Histograms of the normalised thickness profiles $L_{ice}^{*}\left(t\right)$ for all locations (Φ,h) in the samples with BSA solution (**a**), mannitol solution (**b**) and water (**c**).

While investigating the sublimation rate, mainly the radial direction was considered. This rate is reflected in profiles of the ice layer radial thickness $L_{ice}\left(t\right)$ over time. A linear model was fitted to the evolution between the initial thickness $L_{ice}\left(0\right)$ and the last thickness measurement just before the measured end of drying time $t_{end}$ in this linear model, where $L_{ice}\left(t_{end}\right)$ is closest to zero. While this is a major oversimplification of the actual dynamics due to local physical variations, it does offer a useful insight into the 4D data. In Figure 7, a histogram illustrates these profiles normalised over the initial thickness and $t_{end}$, denoted $L_{ice}^{*}\left(t\right)$, for all three samples. It shows that the major part of the radial thickness profiles can be modelled as a linear decline over time. Deviations from the main diagonal indicate accelerating or decelerating decline rates. Some locations show a sudden drop interrupting their linear decline after the measured $t_{end}$. Upon closer investigation, these locations seem linked to a non-radial sublimation front. In the sample containing only water, most profiles didn’t reach a full decline before the end of the scan. A slight upward deviation can be noticed around 20% of the $t_{end}$.

**Figure 8:**
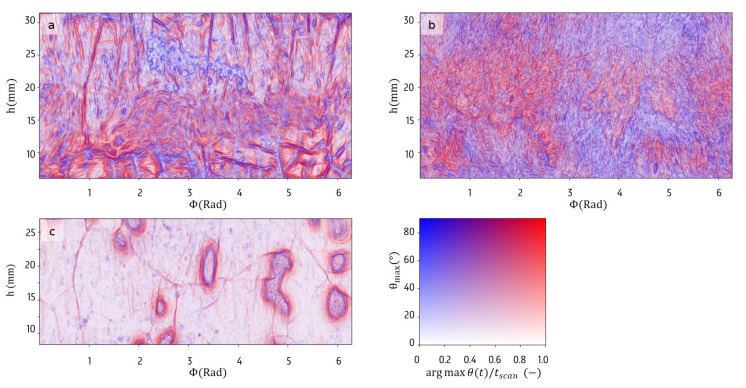
The maps of maximal surface angles with respect to the radial axis for each location (Φ,h) achieved over time show large differences between the BSA solution (**a**), mannitol solution (**b**) and water (**c**). Maximal angles that were measured near the start of the scan are coloured blue, shifting to red for later times.

In the modeling of spin freeze-drying processes, it is typically assumed the surface normal corresponds to the radial direction. As shown in Figure 8, this is not always the case. For each location (Φ,h), the surface normal angle was calculated w.r.t. the radial axis, and the maximal angle in the time series was plotted in these maps along with the time where this maximal value is attained. While the solution with only water is mainly flat throughout the experiment, a few large structures with steep edges are visible. These structures expand over time, as can be deduced from the color scale. The datasets of the BSA solution and mannitol solution display many distinct patterns. While the mannitol displays many smaller and random patterns, the BSA and most definitely the water sample have more large-scale stable features such as cracks and holes. Moreover, the time evolution of the directional angle maps also show that the edges that have large angles with the radial axis also propagate in the direction of these edges upon sublimation. These sharp edges explain the sudden drop in radial thickness encountered above. This complicates modelling of the sublimation process, as it increases the freedom with which these processes occur. The local behaviour depends on the material properties as well as the structural properties of the sample.

**Figure 9:**
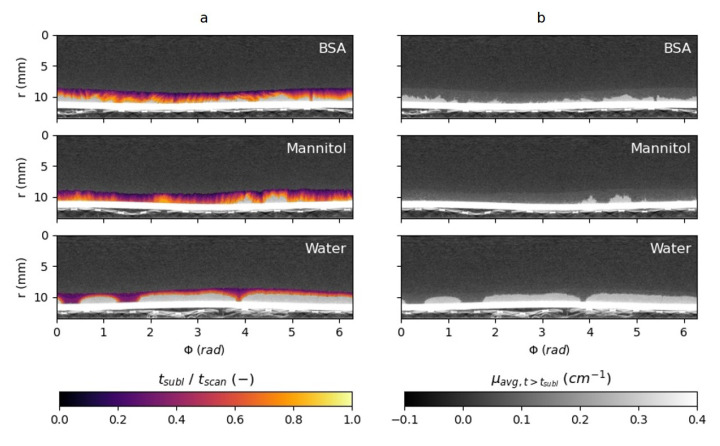
In these horizontal slices, the sublimation time is depicted on the left (**a**). The slices on the right (**b**) show the mean attenuation values after sublimation.

Figure 9 illustrates the sublimation process on a single horizontal slice for all three samples. On the left, the coloured overlay depicts the time of sublimation. For BSA and Mannitol, this leads to these maps with many peaks, mainly along the radial direction. It may be assumed that the direction of sublimation corresponds to the paths on the maps with smooth gradients from the initial scan time to the end time. Discontinuous gradients mark boundaries between distinct sublimation fronts. On the right, the mean attenuation values after sublimation are shown for all locations, the brightness was increased to visualize the faint structures in an attempt to visualize the dry layer. For BSA and Mannitol, clearly a residual structure can be seen where the ice has sublimated. In the pure water sample, this is not visible.

**Figure 10:**
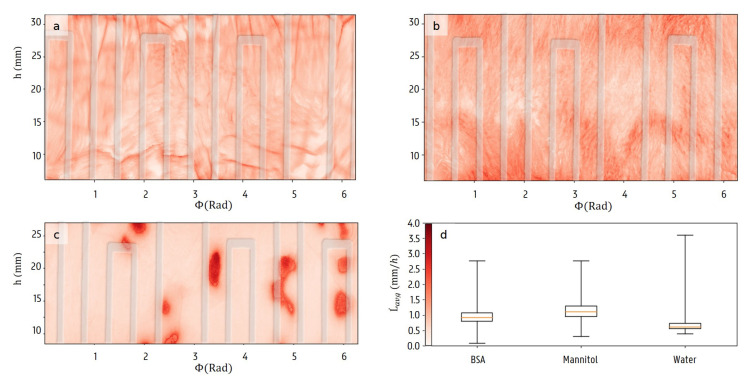
The average radial thickness declines for each location (Φ,h) in the samples with BSA solution (**a**), mannitol solution (**b**) and water (**c**). The heating pad is shown in transparent overlay. The global decline rate statistics are also illustrated in the box plot (**d**).

In Figure 10, the maps of the average radial thickness declines, a proxy for the local average sublimation rate, are shown for all formulations. The heating pad is shown in transparent overlay to check if there is any correlation with the sublimation rates. The water sample displayed some vertically correlated structures with a higher average sublimation rate, but these don’t correspond with the position and spacing of the heating pad. The global average sublimation rates and their standard deviations are 0.94±0.23 mm/h (BSA), 1.14±0.27 mm/h (mannitol) and 0.77±0.42 mm/h (water). The distributions are slightly skewed to higher sublimation rates, but in this context the minimal rates are more important attributes. All ice must have sublimated before the secondary drying phase is initiated, which takes longest in areas that have a high resistance to the sublimation. It does not suffice to take only global measures into account without knowledge of all possible variations within the vial. These large local variations in the sublimation rate are important arguments for modelling the product resistance to a local scale.

## 4. Discussion

### 4.1. Geometrical Sources of Errors

**Figure 11:**
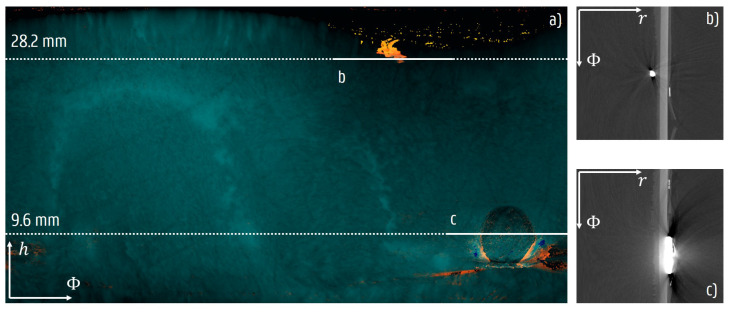
The effect of the thermocouples on the images. Subfigure (**a**) shows the combined thickness *L* and integrated frozen layer thickness $L^{\prime }$ map after 19 min into the scan. The colour scale is identical to Figure 5, where brighter colours reflect a higher thickness and density and orange deviations signal radially interrupted segments. The map was affected significantly by the attached thermocouples that lead to streaks in the reconstructed image. On the horizontal reconstructed slices, it appears that the inner thermocouple, at the top (**b**), is still attached to the ice layer to accurately measure the temperature. The copper plate used to attach the outer thermocouple to the glass vial (**c**) also greatly affected the reconstructed attenuation values and hence the measured thickness of the segmented ice layer.

While the cylindrical symmetry of the sample simplifies the analysis, some regions require special attention as they introduce faults. An important source of errors is formed by the thermocouples attached to measure the ice and vial’s temperature, as illustrated in Figure 11. In an early scan, the copper contact disk around the outer thermocouple obscured the features inside by introducing streaks into the reconstruction. In proceeding scans, this thermocouple was put at the height of the vial bottom, since the temperature within the glass vial was assumed to be approximately constant. There was no direct contact with the ice layer, so the thermocouple corresponded faster to the temperature of the heating pad.

Other analysis artefacts include the geometrical deviations that originated from the vials neck becoming smaller in radius, and from a wrongly aligned cylinder axis. These would affect the measurement of the radial thickness and the surface angles, if not compensated for. In Figure 3, a small misalignment can be noticed that causes the cylindrical glass vial to have slightly different radii at different azimuthal locations. In the case of small deviations, the real radial thickness and surface angles can be assumed to be equal to the actual quantities within the spatial precision of the micro-CT measurement.

The dynamic aspect of the scan introduced additional analysis artefacts related to the geometric stability of the sample. It is important to remain with the same physical location when a dynamic evolution is being profiled. Failing to do this leads to non-physical motion in the sample and thus false observations in the subsequent analysis. In practice, this geometric sample stability was ensured by a scanning system with long-term stability in the hardware components and with accurate records of the geometry. This was investigated in Figure 4. Any remaining global motion that was not related to the sublimation process in the sample was also measured by tracking the clear feature points in the surrounding heating pad. In Figure 10, this unfolded heating pad is depicted. This was monitored to assure the stability along the azimuthal direction.

### 4.2. Motion Artefacts

An important aspect to keep in mind during dynamic scans is the negative effect of the sample’s internal motion on the reconstruction. From Figure 10 it can be seen that the average radial sublimation rates reached up to 180 μm per full rotation of 176.11 s, or approximately 6 voxels. As a consequence, the edges of the ice layer interface to the dry layer were not reconstructed as clearly as static edges. In practice, a smaller sliding window step could be chosen in this continuous acquisition scheme to capture these fast dynamics to some extent. Note that these temporally overlapping reconstructions are only partly independent, and motion artefacts are not restricted to temporal blurring. Indeed, due to the rotation of the CT setup during the scan, the eventual image may display intricate artificial patterns. The state of the sample must be consistent for all projections within the chosen reconstruction window.

It is impossible to improve the temporal resolution using the same setup without affecting the spatial information that is revealed in the reconstruction. However, when the acquisition time of each single projection is decreased, the noise levels increase, masking the contrast between the separate regions of the sample.

### 4.3. Imaging the Dry Layer

Due to the low density of the dry cake layer and the strong attenuation of the ice and the glass vial, this layer could not be observed properly in this data at the set tube accelerating voltage of 90 kVp. Only a hint of the dry layer was observed using the time-averaged reconstructed attenuation coefficients after local sublimation. Other experiments [32] have succeeded in imaging this in a single static scan. Note that the voltages used in those experiments were lower and the scan time per rotation was higher. In later work, this dry layer should be scanned to yield a qualitative comparison of the dynamic sublimation behaviour and the dry layer structure. The dry layer pore network offers insightful information on the driving dynamics of the sublimation. Conversely, the time-resolved structure of the sublimation interface gives a handle on explaining how the dry layer was formed.

### 4.4. Chemical Influence on the Sublimation

In Figure 6 and Figure 8, clear structural differences were noticed between the three samples, that were therefore attributed to their respective chemical composition. Moreover, locally within the samples, very stable features were observed. It is known that the speed of the freezing influences the crystallinity of mannitol within the sample. To ensure a correct interpretation of these structural features, a study of the chemical structure of the end product may offer additional and necessary information. Of course, this cannot be easily performed non-destructively while the primary drying scan is still in process, as such an addition to the setup will further increase the cost and complexity of this scanning technique. However, it is possible to study the structure of the dry cake when it is fully formed, e.g., using X-ray Diffraction (XRD) [40] to this end. A future study may include measuring the crystallinity for different drying speeds to improve the interpretation of the structural differences.

## 5. Conclusions

In this work, the sublimation of spin-frozen vials was imaged with a time-resolving micro-CT scan while accurately tracking the process’ environmental conditions induced in situ. Three different samples (a BSA solution, a mannitol solution and deionised water) were scanned in a continuous acquisition scheme with a temporal resolution down to 1.47 min and voxel sizes of 30 μm.

The symmetry of the vial allowed to greatly reduce the vast amount of data to useful 2D figures that mapped the local parameters of the ice layer surface. Local variations in the sublimation behaviour were noticed, leading to significant differences in the expected end time of primary drying. These variations of the process conditions should be modelled quantitatively, to ensure a stable product that is not affected by residual crystalline water when the secondary drying is initiated.

Previous studies that characterized the final pore structure of a fully freeze-dried cake relied on mathematically challenging models to estimate the drying kinetics. In situ scanning of the sublimation process gives direct access to this information by monitoring the sublimation front in a time-resolved reconstruction. The dried layer could not be visualized in these scans due to a trade-off of the spatial and temporal resolution. In future work, the combination of this dynamic scan and a high resolution scan of the dry layer will offer this valuable information.
